# Evaluation of Flow Rate, pH, Buffering Capacity, Calcium, Total Protein and Total Antioxidant Levels of Saliva in Caries Free and Caries Active Children—An *In Vivo* Study

**DOI:** 10.5005/jp-journals-10005-1034

**Published:** 2009-04-26

**Authors:** AR Prabhakar, Reshma Dodawad, Raju OS

**Affiliations:** 1 Professor and Head, Department of Pedodontics and Preventive Dentistry, Bapuji Dental College and Hospital Davangere-577004, Karnataka, India; 2 Postgraduate Student, Department of Pedodontics and Preventive Dentistry, Bapuji Dental College and Hospital Davangere-577004, Karnataka, India; 3 Professor, Department of Pedodontics and Preventive Dentistry, Bapuji Dental College and Hospital, Davangere-577004 Karnataka, India

**Keywords:** Saliva, flow rate, pH, buffering capacity, calcium, protein, total antioxidant capacity, dental caries.

## Abstract

*Background and objectives:* The purpose of this study was to evaluate the relationship between the physicochemical properties of saliva such as flow rate, pH, buffering capacity, calcium level, total protein and total antioxidant levels in caries free and caries active children.

*Materials and methods:* The present study included one hundred and twenty healthy children who were divided into two groups and subdivided according to gender. They were further divided into caries free and caries active children with 15 children in each group. Unstimulated saliva was collected by suction method and flow rates were determined. The samples were then analyzed for pH, buffering capacity, total protein, calcium and total antioxidant levels. The data was then statistically analyzed using student’s ‘t’ test {unpaired}.

*Results:* The results revealed that when all these parameters were compared among the caries free and caries active children, the flow rate, pH and buffering capacity were slightly reduced in caries active children, but the total protein and total antioxidant capacity of saliva increased significantly in caries active children and the total calcium decreased significantly in caries active children.

*Conclusion:* Within the limitation of this study, we can conclude that, the physicochemical properties of saliva play a major role in the development of caries.

## INTRODUCTION

Among the oral diseases, dental caries is the most common chronic disease of mankind.^1^ It affects all people regardless of their sex, socioeconomic strata, race and age. It is also profoundly affected by other factors like oral hygiene and saliva.^2^

The saliva circulating in the mouth at any given time is termed as whole saliva and it comprises of a mixture of secretions from the major and minor salivary glands and traces from the gingival crevicular fluid. Saliva definitely promotes oral health and hence lack of its secretion contributes to the disease process.^2^,^3^ The saliva by constantly bathing the teeth and oral mucosa, functions as a cleansing solution, a lubricant, a buffer and an ion reservoir of calcium and phosphate which are essential for remineralization of initial carious lesions.

Recently, it has been claimed that the imbalances in levels of free radicals, reactive oxygen species and antioxidants in saliva may play an important role in the onset and development dental caries.^4^ Hence, evaluation of those factors in saliva that may increase the risk of individuals to dental caries, can pave way to make recommendations that will cater specifically to needs of an individual.^5^

## MATERIALS AND METHOD

One hundred and twenty children of age group between 7-14 years reporting to the Department of Pedodontics and Preventive Dentistry, Bapuji Dental College and Hospital, Davangere, were included as subjects of this study. They were divided into two groups; Group-I and Group-II comprising of age groups 7 to 10 and 11 to 14 years respectively. Both the groups were then subdivided according to gender with 30 children in each group. They were then further divided into caries active and caries free groups, with 15 children in each group.

The inclusion and exclusion criteria used are as follows:

### Inclusion Criteria

- Free from systemic or local diseases which affect salivary secretions.
- Children should be permanent residents of Davangere city and should be consuming only municipal water.
- Caries status was assessed according to the WHO criteria:
  - Caries active children having at least five decayed tooth surfaces.
- Caries free children having no caries, DMFS = 0.

### Exclusion Criteria

- Patients who are physically and medically compromised.
- Patients who are on medications.
- Patients who have arrested carious lesions.

Stratified randomized sampling procedure was employed for the statistical analysis.

Unstimulated saliva was collected for the study. The saliva was allowed to accumulate in patient’s mouth for 2 minutes and was aspirated directly from the floor of the mouth. Each sample was estimated for pH, buffering capacity, total calcium, total protein, and total antioxidant capacity.

## SALIVARY ANALYSIS

### Estimation of Flow Rate of Saliva

The flow rate of saliva was estimated by asking children to spit into (the) preweighed plastic cylinders for 5 minutes. These plastic cylinders (containing saliva) were then weighed and the flow rate was calculated in gm/ml which is almost equivalent to ml/min.^6^,^7^

### Estimation of pH and Buffering Capacity

pH of saliva was measured by using manual pH meter.

*Buffering capacity of saliva (by Ericsson method 1959):* 0.5 ml of saliva was added to 1.5 ml of 5 mmol/l HCl. The mixture was vigorously shaken and then centrifuged for one minute and allowed to stand for 10 min when the final pH was measured by using manual pH meter.^8^

### Estimation of Total Calcium and Total Protein of Saliva

Estimation of total protein and calcium was done by autoanalyzer which works on the principle of atomic absorption spectrophotometer.^8^ (Total calcium and total protein was estimated). The estimation was done by using ‘Human diagnostic kit’ {Germany}.

### Estimation of Total Antioxidant Capacity of Saliva

Total salivary antioxidant levels were estimated by using a spectrophotometer.^8^

## STATISTICAL ANALYSIS

Results are presented as mean ± standard deviation values. Student’s ‘t’ test was used to compare the mean values between caries free and caries active groups.

A ‘P’ value of 0.05 or less was considered for standard significance.

## RESULTS

On observation flow rate, pH and buffering capacity are slightly decreased in caries active children compared to caries free children but the total protein and total antioxidant capacity of saliva increased significantly and total calcium decreased significantly in caries active children (Table 1).

**Table 1:** TABLE 1: Salivary parameters in caries active and caries free children

*Gender*		*Age*		*Caries*		*Flow rate*		*pH*		*Buffer capacity*		*Total protein*		*Total calcium*		*Total*
				*activity*												*antioxidants*
Girl		7-10		CF		3.78 ± 0.74		7.15 ± 0.15		5.44 ± 0.43		5.68 ± 1.33		8.66 ± 1.66		0.16 ± 0.03
Girl		7-10		CA		3.46 ± 0.43		7.07 ± 0.43		5.32 ± 0.40		6.61 ± 1.30		8.08 ± 2.09		0.23 ± 0.05
	CF versus CA					t = 1.44		t = 0.62		t = 0.79		t = 1.93		t = 0.84		t = 4.18
						p = 0.16		p = 0.54		p = 0.44		p = 0.06		p = 0.41		p < .001
Boy		7-10		CF		3.83 ± 0.84		7.28 ± 0.34		5.37 ± 0.44		5.59 ± 1.34		8.90 ± 1.39		0.16 ± 0.05
Boy		7-10		CA		3.76 ± 1.18		7.24 ± 0.45		5.03 ± 0.76		7.23 ± 1.37		7.41 ± 1.89		0.20 ± 0.04
	CF versus CA					t = 0.18		t = 0.28		t = 1.47		t = 3.32		t = 2.46		t = 2.91
						p = 0.86		p = 0.78		p = 0.15		p < 0.01		p < 0.05		p < 0.05
Girl		11-14		CF		4.28 ± 1.51		7.17 ± 0.31		4.76 ± 0.44		5.57 ± 1.19		9.11 ± 1.28		0.19 ± 0.04
Girl		11-14		CA		4.25 ± 1.18		7.15 ± 0.39		5.19 ± 0.47		6.57 ± 1.50		7.97 ± 1.57		0.23 ± 0.04
	CF versus CA					t = 0.07		t = 0.16		t = 0.79		t = 2.56		t =2.18		t = 2.90
						p = 0.95		p = 0.88		p = 0.44		p <0.05		p <0.05		p < .001
Boy		11-14		CF		4.35 ± 1.05		7.47 ± 0.38		4.91± 0.55		5.28 ± 0.84		9.13 ± 1.02		0.19 ± 0.04
Boy		11-14		CA		4.58 ± 1.17		7.31 ± 0.21		4.90 ± 0.41		7.36 ± 1.52		7.12 ± 1.60		0.22 ± 0.04
	CF versus CA					t = 0.56		t = 1.36		t = 0.07		t =4.63		t =4.10		t = 2.21
						p = 0.58		p = 0.19		p = 0.94		p < 0.01		p < 0.01		p > .005
Unpaired ‘t’ test																
p < 0.05 Significant																
p > 0.05 Not significant																

## DISCUSSION

Under resting conditions without the exogenous stimulation that is linked with feeding, there is a slow flow of saliva which keeps the mouth moist and lubricates the mucous membrane. This unstimulated flow, is what is secreted by the salivary glands the majority of the time. Unstimulated saliva is essential for the health and wellbeing of the oral cavity and also bestows a strong protective effect to the oral cavity, against dental caries.^9^,^10^

In general, higher the flow rate, faster the clearance and higher the buffer capacity and thus lesser microbial attacks.^11^

The results of our study showed that the salivary flow rate was decreased in caries active in comparison to caries free children but, was not statistically significant. Parallel results were seen in the studies conducted by Browne et al and Scully where they showed that dental caries is probably the most common consequence of hyposalivation.

In contrast to the above, the studies conducted by Birkhed, Heintze, and Russell et al reported that there was no correlation between salivary secretion rate and caries activity.^11^

In relation to pH, the outcome of the present study showed that in caries active children pH ranged from 6.20 to 7.90. It has been well documented that the dissolution of enamel occurs when the pH falls below critical pH, i.e. 5.5, so the values obtained in the study are not adequate to cause demineralization of inorganic substance of the tooth.

The study showed that pH and buffering capacity had a weak correlation with caries activity. Hence, it can be speculated that other factors like micro flora, diet, and retention of food might have dominated the buffering capacity to initiate caries, which is a multifactorial diesease.^12^ Similar results were seen in a study conducted by Tuhunoglu OS who showed no correlation between pH values and caries activity regardless of the age and gender.

They were dependent upon individual and environmental variations.^8^

The results of the current study was in contrast to the study conducted by Ericsson which showed that salivary buffering capacity has a negative relationship with caries incidence.^13^

In the present study, the mean calcium concentration in caries active children was decreased compared to caries free children. The decrease in the caries experience in children with high calcium concentration in saliva is attributed to the process of remineralization of the incipient caries lesions. The saliva which is supersaturated with calcium and phosphate acts as a reservoir for these essential ions.^10^ In such a conducive environment the process of remineralization overrides demineralization.

With regard to the total protein level, the study showed that this variable increased in caries active children in comparison to caries inactive.

The higher total antioxidant values in caries active children as found in the study can be attributed to elevated protein levels. In a previous study it was suggested that the major antioxidant in saliva was, urate thus it can be concluded that salivary antioxidant levels must be in a linear association with total protein levels. Our results were in sync with a related study conducted by Tulunoglu.O.S that also reported that total antioxidant capacity of saliva increased with caries activity.^10^

## CONCLUSION

Dental caries is a complex and dynamic process where a multitude of factors influence and initiate the progression of disease. One of the most important factor which influences the development of dental caries is saliva. The physicochemical properties of saliva like pH, buffering capacity, salivary flow rate, concentration of various components like proteins, calcium and antioxidant defense system play a major role in the development of caries.

Within the precincts of this study it was established that total antioxidant capacity of saliva has a linear relation with caries, i.e. as the severity of caries increased, the TAC levels also increase. But in order to extrapolate these findings, studies using larger sample size are needed.
